# Mycoplasma Pneumoniae-Induced Rash and Mucositis: A Systematic Review of the Literature

**DOI:** 10.51894/001c.25284

**Published:** 2021-08-30

**Authors:** Daniel Lofgren, Christopher Lenkeit

**Affiliations:** 1 Graduate Medical Education, Otolaryngology - Head & Neck Surgery Resident McLaren Oakland Hospital, Pontiac, MI, USA

**Keywords:** mycoplasma pneumoniae, mycoplasma pneumoniae-induced rash and mucositis, mucositis, rash, mirm, mim

## Abstract

**INTRODUCTION:**

*Mycoplasma pneumoniae* (MP) is a common respiratory pathogen that can result in community-acquired pneumonia (CAP). Approximately 25% of patients diagnosed with MP experience extrapulmonary manifestations. *Mycoplasma*-induced rash and mucositis (MIRM) was coined as a unique disease process in 2014. MIRM has prominent mucositis with or without a characteristic vesiculobullous and/or atypical targetoid eruption. Appropriate identification of this disease is important because it has a milder disease course with low rates of sequelae, and lower mortality compared to Stevens-Johnson syndrome, erythema multiforme, and toxic epidermal necrolysis. The objective of this systematic review was to examine the English literature on *Mycoplasma Pneumonia*-induced rash and mucositis since the establishment of its diagnosis in 2014.

**METHODS:**

The following online databases were used to identify appropriate studies that met the established inclusion and exclusion criteria: Pubmed, Cochrane, MedLine, Health Evidence, EPPI center, Allied Health Evidence. The following MesH search terms were used to further identify articles; “*Mycoplasma pneumoniae* induced rash and mucositis,” “*Mycoplasma pneumoniae* rash and mucositis,” “*Mycoplasma pneumoniae* rash,” “*Mycoplasma pneumoniae* mucositis,” “MIRM,” “*Mycoplasma* induced rash and mucositis,” “*Mycoplasma* rash and mucositis,” “*Mycoplasma* rash,” and “*Mycoplasma* mucositis.” Data was extracted following the Preferred Reporting Items for Systematic Reviews and Meta-Analyses (PRISMA) guidelines.

**RESULTS:**

One hundred and seventy-five records were initially screened, and nineteen studies were included in the review, leading to a total of 27 patients. Patients had a mean age of 16 years old (Range 4 - 46 years old), with the majority being males (74%). Pulmonary symptoms tended to precede extrapulmonary symptoms on an average of 7.8 days. Extrapulmonary symptoms consisted of oral lesions (96.3%) followed by ocular lesions (92.6%) and genital lesions (59.3%). Female patients were more likely to have genital lesions (71.4%) when compared with male patients (55%). Cutaneous rashes occurred in approximately one-half of the patients, which supports the theory that MIRM is a separate clinical entity from SJS and other related skin disorders.

Confirmatory testing for MIRM was performed using IgM/IgG *Mycoplasma* antibody testing or PCR in 19 (66.7%) and 6 (22.2%) patients respectively, although four cases reported the use of both serology and PCR, while five did not report confirmatory testing. Systemic antibiotics were used frequently in treatment 22 patients (77.8%) and 27 (100%) of the patients received various supportive care. Approximately 11 (37%) patients of reported cases used systemic steroids to reduce systemic inflammation. Other systemic treatments were used in six (21.4%) cases, and included intravenous immunoglobulins and cyclosporine A. Only eight patients (22.2%) reported having any lasting sequelae.

**CONCLUSION:**

*Mycoplasma*-induced rash and mucositis is a recently described extra-pulmonary manifestation of *Mycoplasma pneumoniae* infections. To the best of the authors’ knowledge, this is the first systematic review of the MIRM literature since the introduction of the diagnosis in 2014. The authors hope that this review can serve to better our current understanding and lead to improved identification, work-up, and treatment of this disease. One notable limitation of this study is the relatively small sample size, which is due to the recent introduction of the term.

## Introduction

*Mycoplasma pneumoniae* (MP) is a common respiratory pathogen that can result in community-acquired pneumonia (CAP).^1^ One 2016 meta-analysis reported MP’s prevalence as 10.1% of all CAP, with higher rates in children (17.6%) compared to adults (7.2%).^1^ Approximately 25% of patients diagnosed with MP experience extrapulmonary manifestations, which include pericarditis (i.e., inflammation of pericardium), thrombosis (i.e., blood clot), hepatitis (i.e., liver inflammation), hemolytic anemia (i.e., destruction of red blood cells), arthritis (i.e., inflammation of joints), encephalitis (i.e., inflammation of brain), glomerulonephritis (i.e., inflammation of kidneys), mucositis (i.e., mucosal inflammation), and varying dermatologic manifestations.^2–6^ Historically, reported dermatologic manifestations of *Mycoplasma pneumoniae* were considered to be on the spectrum of erythema multiforme (EM), Steven-Johnson-Syndrome (SJS), and toxic epidermal necrolysis (TEN).^3^ EM is a skin immune rection appearing as raised red rashes in many different shapes, versus SJS which consists of a painful rash that blisters and sheds skin over body and mucous membranes. Toxic epidermal necrolysis is a more severe form of SJS, covering more surface area of the body.^3^ One smaller retrospective review of 30 pediatric patients looked for possible etiologies of EM and found that over 13.3% tested positive for MP.^7^

Although historically MP related mucocutaneous disease has fallen within the spectrum of EM, SJS, and TEN; recent literature has proposed that it be to be its own separate disease process. Canavan and colleagues performed the largest systematic review to date and were the first to coin the term *Mycoplasma*-induced rash and mucositis (MIRM) as a unique disease process in 2014.^2^ This was based on their analysis of 202 cases of mucocutaneous (mucous membrane and skin) disease in patients that tested positive for *Mycoplasma pneumoniae*. Clinically, Canavan et al noted a varying degree of mucosal involvement with or without cutaneous involvement. They found a distinct disease morphology that did not fit into the established EM, SJS, and TEN diagnoses. MIRM has prominent mucositis (mucosal inflammation) with or without a characteristic skin vesicles and/or atypical target shaped eruption that one might see in the SJS spectrum. MIRM also generally has a milder disease course with low rates of sequelae, and lower mortality compared to EM, SJS, and TEN. Other studies have noted that the pathophysiology and treatment of this distinct clinical entity differs from previously described Mycoplasma induced erythema multiforme.^2,4,7^

## Pathophysiology

The pathophysiology of MIRM is still not fully understood and many theories have been proposed. The most widely accepted theory suggests cloning of B cells with cutaneous immune complex deposition and complement formation causes extrapulmonary symptoms.^2,4^ Molecular mimicry between mycoplasma’s adhesion molecules and keratinocyte (i.e., skin cell) antigen has also been proposed but is less widely accepted.^2,4,5,8,9^ There is minimal information regarding histology of the mucocutaneous lesions that have been reported in the literature. Cutaneous leukocytoclastic vasculitis (i.e., inflammation of small capillary vessels) has been associated with *Mycoplasma pneumonia* in the past and is characterized by neutrophilic perivascular infiltrate around the lesional sites.^3^ Amode et al. described a Toxic Epidermal Necrolysis-like histologic pattern consisting of minimal dermal change with intense and keratinocyte apoptosis in 14 patients with MIRM.^10^

## Epidemiology and Symptomatology

Generally, patients suffering from MIRM are afflicted in the winter months, are male (60-66%), young (8.7 to 11.9 years old), and experience prodromal (i.e., non-specific) symptoms including fever, malaise, and cough on average 7-10 days before mucocutaneous symptoms.^2,4,8,10^ In the original description, Canavan and colleagues noted sparse cutaneous involvement - defined as a few scattered lesions - in 47% of patients, compared with severe mucositis alone (34%) and moderate cutaneous involvement alone (19%). Cutaneous lesions of the extremities (47%) were more common than lesions of the trunk (23%) and generalized involvement (31%).^2,10^ Reported cutaneous lesions varied in appearance from vesiculobullous (77%) to targetoid (48%), papular (14%), macular (12%), and morbilliform (9%).^2,4,5^ One prospective cohort study of 152 children with CAP by Sauteur et al, revealed 44 patients (28.9%) tested positive for MP, and of these children, ten (22.7%) developed mucocutaneous lesions. Of these ten patients, five developed maculopapular skin eruptions (11.4%), two had urticaria (4.5%), and three patients had mucocutaneous disease (6.8%).^8^

The involvement of mucosal surfaces appears to be the hallmark associated with this disease. The oral cavity was involved in 94% of patients with symptoms ranging from erosions and ulcers to denuded tissue.^2,4,5^ Ocular involvement was the second most common extrapulmonary symptom occurring in 82% of patients.^2,4^ Patients presented with purulent bilateral conjunctivitis, photophobia, pseudomembrane formation, ulceration, and eyelid edema.^2,4,5^ Urogenital lesions occurred in approximately 63% of patients. Interestingly, in Canavan’s study, only four of the 202 patients did not have mucosal involvement.^2,4,5,11^

## Diagnosis

Diagnosis of MIRM has historically been based on positive identification of *Mycoplasma* *pneumoniae* on clinical, radiological, and laboratory findings with associated extrapulmonary symptoms. Generally, patients are admitted to the hospital for symptoms of pneumonia and work up confirms the diagnosis of MP. Diagnosis is generally made with serologic testing using cold agglutinins, bullae cultures, and polymerase chain reaction (PCR), but more recently, some authors report using enzyme-linked immunoassays (ELISA) and *Mycoplasma pneumoniae* IgM antibody levels.^2,9^ Laboratory findings can include elevated c-reactive protein (CRP), erythrocyte sedimentation rate (ESR), and leukocytosis with left shift.^4,5,8,9,12,13^ Other pathogenic causes of mucocutaneous lesions must be ruled out including Herpes Simplex Virus, Cytomegalovirus, Varicella-Zoster, *Chlamydia* species, Coxsackie virus, influenza B, *Staphylococcus aureus*, and a host of other autoimmune diseases.^4,7,13,14^

The proposed diagnostic criteria for classic MIRM includes clinical and laboratory evidence of atypical pneumonia caused by *Mycoplasma pneumoniae* with the following: ≥ 2 involved mucosal sites, less than 10% involved cutaneous surface area, few vesiculobullous lesions or atypical scattered targets with or without targetoid lesions. There are two proposed variants of MIRM called severe MIRM with extensive involvement of atypical targetoid lesions or blisters and MIRM sine (without) rash, which showed minimal morbilliform lesions with few vesicles.^2,4,14^ Interestingly, patients presenting with MIRM sine rash had higher rates of mucosal involvement: oral (100%), ocular (92%), and urogenital (78%).^2,4^

Since the establishment of the MIRM classification system in 2014, there have been no comprehensive reviews of reported MIRM studies in the literature. The authors wish to systematically review the current literature to provide an up-to-date and comprehensive picture of the presentation, diagnosis, work-up, and treatment of this relatively uncommon disease.

## Methods

**Figure 1.**
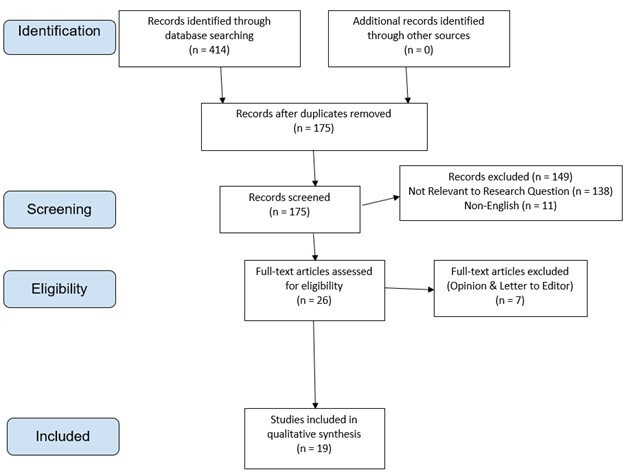
Article Inclusion directed by Preferred Reporting Items for Systematic Reviews and Meta-Analyses (PRISMA) guidelines.

This study was designed as a systematic review of the literature of MIRM since the establishment of the diagnosis in 2014. The article selection process used can be seen in Figure 1, which follows the Preferred Reporting Items for Systematic Reviews and Meta-Analyses (PRISMA) guidelines. Inclusion criteria included any study type from January 2014 to April 2020 containing adult and/or pediatric patients who were clinically diagnosed with MIRM. English language or English translated papers were included, and the authors were required to have full access to abstract and manuscript. Exclusion criteria involved studies that were determined by the authors to be letters to the editor or opinion pieces. The following online databases were used to identify appropriate studies that met our inclusion and exclusion criteria: Pubmed, Cochrane, MedLine, Health Evidence, EPPI center, Allied Health Evidence. The following MesH search terms were used to further identify articles “*Mycoplasma* *pneumoniae* induced rash and mucositis,” “*Mycoplasma* *pneumoniae* rash and mucositis,” “*Mycoplasma* *pneumoniae* rash,” “*Mycoplasma pneumoniae* mucositis,” “MIRM,” “*Mycoplasma* induced rash and mucositis,” “*Mycoplasma* rash and mucositis,” “*Mycoplasma* rash,” and “*Mycoplasma* mucositis.”

All abstracts for the studies were screened and reviewed by the lead authors (DL, CL) to determine if the studies met the inclusion or exclusion criteria above. For data collection, all studies were reviewed independently by all authors and added to a common Microsoft excel sheet with title and author and abstract. Duplicates were then removed after all databases were searched thoroughly using the terms above. The author team independently reviewed each paper to determine the significance of the topic and make sure that they met the current established criteria for MIRM. All authors followed the above criteria. Statistical results were reported in both numbers and percentages.

## Results

A thorough review of the literature from January 2014 to April 2020 revealed 19 studies with a total of 27 patients who met clinical criteria for MIRM since it was clarified diagnostically in 2014. All studies meeting the above inclusion criteria were case reports. Biological gender, age, pulmonary symptoms, extrapulmonary symptoms, diagnostic testing, results of chest imaging, treatments, and outcomes were recorded in Table 1. Patients ranged in age from 4 to 46 years of age with a mean age of 16. Of the total patient population, 20 (74%) patients were males. Symptoms, diagnosis, treatment, and sequelae can be seen in Table 2. Pulmonary symptoms varied from mild dyspnea and cough to severe respiratory distress.

**Table 1.** Age and gender demographics for the patients included in this study

**Category**	**Number of Patients (Percentage/SD)**
**Total Patients**	27
**Male Patients**	20 (74.1%)
**Female Patients**	7 (25.9%)
**Mean Age (Years Old)**	16.3 (±9.95)
**Mode Age (Years Old)**	16
**Median Age (Years Old)**	14
**Range (Years Old)**	4-46

The results of imaging also varied which is expected given MP’s designation as an atypical pneumonia. Pulmonary symptoms tended to precede extrapulmonary symptoms by an average of 7.8 days. The most common extrapulmonary symptoms were oral lesions in 26 (96.3%) patients followed by 25 patients with ocular lesions (92.6%) and 16 (59.3%) with genital lesions. Interestingly, female patients were more likely to have genital lesions, 5 (71.4%), when compared with male patients, 11 (55%). Cutaneous rashes occurred in approximately half of the patients, which supports that MIRM appears to be a separate clinical entity from SJS and other related skin disorders. Diagnosis of *Mycoplasma pneumoniae* was done either clinically or with chest radiography in all cases. Confirmatory testing was performed using IgM/IgG *Mycoplasma* antibody testing or PCR in 19 (66.7%) and 6 (22.2%) patients respectively, although 4 cases reported the use of both serology and PCR, and 5 did not report confirmatory testing.

Twenty-two patients were given systemic antibiotics for treatment (77.8%) and 27 (100%) of the patients received supportive care, which included intravenous hydration, pain control, and topical medications for localized wounds (steroids or antibiotics). Approximately 11 (37%) reported cases used systemic steroids to reduce systemic inflammation. Other systemic treatments included either intravenous immunoglobulins and cyclosporine A in 6 (21.4%) cases. Only eight patients (22.2%) reported having any lasting sequelae. Two patients noted hypo/hyperpigmentation changes, two patients noted ocular scarring and xerophthalmia, one patient had persistent cutaneous pain, another patient had chronic phimosis, and the final two patients noted residual and recurrent skin lesions.

## Discussion

Although there are no standardized treatment guidelines for patients suffering from MIRM supportive management is the mainstay of treatment, which includes pain management, intravenous hydration, and mucosal care.^2,4,8,9,15^ Overall, our study data reports that twenty-two (77%) patients were treated with antibiotics, 11 (37%) with corticosteroids, and 11% with IVIG. No high-powered studies have compared resolution times or efficacy of treatments for MIRM. Recently, a case series of three patients with MIRM treated with cyclosporine A (CsA) reported a significantly shortened duration of hospital admission and morbidity if given within 48 hours of mucocutaneous eruption. Their patients stayed in the hospital for 5-7 days compared to the previously reported length with supportive care of 11-14 days. This appeared to translate into cheaper costs and less infectious disease risk when compared with IVIG alone.^14^

Some patients may require increased levels of care including intensive care unit or burn center management.^2,4^ Sauteur and colleagues noted a statistically significant increase in hospital stay length in patients suffering from MIRM compared with non-mycoplasma EM patients (9.5 vs. 5.1 days) with a reported odds ratio (OR) of 9 (95% CI, 1.4-81.4; P = 0.01). They also reported increased oxygen requirements in patients with MIRM vs. CAP alone (OR = 17.6; 95% CI, 1.5-984.1; P = .007).^8^ The recurrence rate of MIRM has been reported as 8%, while the mortality rate has been noted as 3%, with all reported deaths prior to 1940.^2^

Although the majority of MIRM patients are known to generally make a full recovery (81%), a variety of complications have been noted in the literature.^2^ Compared with CAP, patients suffering from MIRM are more likely to develop long term sequelae.^8^ Orbital complications are noted in approximately 9% of patients and include conjunctival shrinkage, corneal ulceration, blindness, ocular synechiae, lash loss, and xerophthalmia (i.e., dry eyes). Postinflammatory pigmentation changes are noted in about 5.6% of cases.^2,8^ Oral and genital synechiae each occur in approximately 1% of patients. Other reported rare complications include genital adhesions, hematemesis (i.e., bloody emesis), epiglottitis (i.e., inflammation of the epiglottis), subcorneal pustulosis (i.e., pustules of the eye), B-cell lymphopenia (i.e., low B-Cell count), and death.^2,4,5,10^ Genetic susceptibility has also been theorized to play a role due to the reported 8% recurrence rate and distribution in families as reported in the literature.^2,6^

**Table 2.** Symptoms, diagnosis, and treatment of patients suffering from MIRM

**Category**	**Oral Symptoms**	**Ocular Symptoms**	**Genital Lesions - All Genders**	**Genital Lesions - Male Patients**	**Genital Lesions - Female Patients**	**Cutaneous Rash**	
Number of Patients	26	25	16	11	5	15	
Percentage (%)	96.3	92.6	59.3	55.0	71.4	55.6	
	**Seropositivity (IgM/IgG)**	**Polymerase Chain Reaction (PCR)**	**Systemic Antibiotics (PO/IV)**	**Systemic Steroids (PO/IV)**	**Intravenous Immunoglobulins (IVIG)**	**Cyclosporine**	**Sequelae**
Number of Patients	19	6	22	11	3	3	6
Percentage (%)	66.7	22.2	77.8	37.0	10.7	10.7	22.2

The authors recognize that this study has inherent limitations. Due to the recent establishment of the diagnosis and the similar symptomology and mimicry to other skin disorders like TEN, SJS, and EM, MIRM has a limited number of published cases. This study provides the only comprehensive review of all of the cases reported in the literature since inception of the diagnosis, which will allow for further studies to compare their data. Another limitation of this study was the use of English only or English translated literature only. Unfortunately, all manuscripts included in the literature were either case reports or case studies and have inherently no power, so it’s hard to draw high powered statistical evidence from them. Additionally, since our data is largely drawn from case studies there is some missing information regarding patients, including diagnostic testing, further description of supportive treatment, and cutaneous rash onset. This makes it difficult to standardize possible diagnostic and treatment protocols.

## Conclusion

*Mycoplasma*-induced rash and mucositis is one of the more recently discovered extrapulmonary manifestations of *Mycoplasma pneumoniae* infections. The study is the first systematic review of the MIRM literature since the introduction of the diagnosis in 2014. The authors hope that this review can serve to better our current understanding of this disease and lead to improved identification, work-up, and treatment of this disease.

### Disclosure/COI

The authors declare that they have no conflicts of interest to disclose
